# Draft Genome Sequence of the Yeast *Starmerella bacillaris* (syn., *Candida* *zemplinina*) FRI751 Isolated from Fermenting Must of Dried Raboso Grapes

**DOI:** 10.1128/genomeA.00224-17

**Published:** 2017-04-27

**Authors:** Wilson José Fernandes Lemos Junior, Laura Treu, Vinícius da Silva Duarte, Stefano Campanaro, Chiara Nadai, Alessio Giacomini, Viviana Corich

**Affiliations:** a Department of Agronomy, Food, Natural Resources, Animals and Environment, University of Padova, Legnaro, Italy; b Department of Microbiology, Universidade Federal de Viçosa, Viçosa, Brazil; c Department of Biology, University of Padova, Padua, Italy; d Department of Environmental Engineering, Technical University of Denmark, Kongens Lyngby, Denmark; e Interdepartmental Centre for Research in Viticulture and Enology, University of Padova, Conegliano, Italy

## Abstract

*Starmerella bacillaris* is an ascomycetous yeast commonly present in enological environments. Here, we report the first draft genome sequence of *S. bacillaris* FRI751, which will facilitate the study of the characteristics of this interesting enological yeast.

## GENOME ANNOUNCEMENT

The ascomycetous yeast *Starmerella bacillaris* (syn., *Candida zemplinina*) is frequently found in spontaneous must fermentation, usually at a relatively high population level of 10^4^ to 10^6^ cells/ml (1), in grape marcs (2), and it is also normally present on botrytized grapes.

This species was isolated for the first time in Napa Valley (CA) in 2002 (3), and 1 year later, Sipiczki (4) assigned this *Candida* sp. to a novel species under the name *Candida zemplinina*, due to the significant differences observed in the rRNA sequence from that of the related species *Candida stellata* (5). For a long time, *C. zemplinina* has been confounded with its close species *C. stellata*, which shares similar ecological niches, particularly in grape and wine environments. Finally, it was established as *Starmerella bacillaris* (6).

*S. bacillaris* is able to ferment glucose, sucrose, and raffinose but not galactose, maltose, or lactose (6). Unable to grow in vitamin-free medium, it develops well in the presence of high glucose concentration, up to 50% (wt/vol) (6). It is highly fructophilic and a high-glycerol producer (7).

*S. bacillaris* is a psichrotolerant and osmotolerant species (4), and among the non-*Saccharomyces* yeasts of enological interest, *S. bacillaris* is considered one of the most promising species to satisfy modern market and consumer preferences. In particular, it produces less ethanol from must fermentation than *Saccharomyces cerevisiae*, low levels of biogenic amines, and average volatile acidity (8). It is also being tested in association with *Saccharomyces cerevisiae* in mixed or sequential fermentations to reduce alcohol content and to increase the organoleptic properties of wines (7), and its possible use in the vineyard as an antifungal agent against *Botrytis* is under study (8).

In this work, the first genome sequence for an *S. bacillaris* strain is released. Strain FRI751 was isolated from fermentation of dried grapes of Raboso wine, a vine variety cultivated mainly in the Northeast of Italy for the production of passito wines.

*S. bacillaris* FRI751 genomic DNA was prepared by zymolyase digestion, followed by standard phenol-chloroform extraction, as described by Vaughan-Martini and Martini (9). The genome sequence was generated using an Illumina NextSeq 500 platform (1-kb mate-pair libraries) at the Ramaciotti Centre, Sydney, Australia. The sequencing generated 45-fold coverage with 1,435,554 paired-end (2 × 150 bp) and 102,368 unpaired reads (after quality filtering) that were used for the *de novo* assembly by SPAdes 3.10 software (10) (with option -k 21,33,55,77,99,127). The genome size of *S. bacillaris* FRI751 was 9.3 Mbp, divided into 106 contigs longer than 100 bp (*N*_50_ length, 208,744 bp), and the G+C content was 39.4%. Protein-coding gene (CDS) prediction was performed using GeneMark-ES (11) and resulted in 4,028 CDSs and a total of 4,315 exons. Gene annotation was obtained combining two strategies: (i) BlastKOALA (12) was used to search against a nonredundant set of KEGG genes, selecting Saccharomycetaceae as the taxonomy group; and (ii) RPS BLAST was used to compare protein sequences with Eukaryotic Orthologous Groups of proteins (KOG) (13).

The data reported here represent a useful resource to increase the knowledge of *S. bacillaris* metabolism and of its potential technological characteristics as applied to enology.

### Accession number(s).

The whole-genome shotgun project of *S. bacillaris* FRI751 has been deposited in DDBJ/ENA/GenBank under the accession no. MWSF00000000. The version described in this paper is the first version, MWSF01000000.
